# A randomized phase II trial evaluating the addition of low dose, short course sunitinib to docetaxel in advanced solid tumours

**DOI:** 10.1186/s12885-020-07616-4

**Published:** 2020-11-17

**Authors:** Yvonne L. E. Ang, Gwo Fuang Ho, Ross A. Soo, Raghav Sundar, Sing Huang Tan, Wei Peng Yong, Samuel G. W. Ow, Joline S. J. Lim, Wan Qin Chong, Phyu Pyar Soe, Bee Choo Tai, Lingzhi Wang, Boon Cher Goh, Soo-Chin Lee

**Affiliations:** 1 Department of Haematology-Oncology, National University Cancer Institute, National University Health System, Level 7, NUHS Tower Block, 1E Kent Ridge Road, Singapore, 119228 Singapore; 2 University of Malaya Medical Centre, Kuala Lumpur, Malaysia; 3 Cancer Science Institute, National University of Singapore, Singapore, Singapore; 4 The N.1 Institute for Health, National University of Singapore, Singapore, Singapore; 5 Yong Loo Lin School of Medicine, National University of Singapore, Singapore, Singapore; 6 OncoCare Cancer Centre, Singapore, Singapore; 7 Saw Swee Hock School of Public Health, National University of Singapore, Singapore, Singapore

**Keywords:** Tumour vasculature, Anti-angiogenic, Short-course sunitinib, Advanced solid tumours, Docetaxel

## Abstract

**Background:**

We previously reported that low-dose, short-course sunitinib prior to neoadjuvant doxorubicin-cyclophosphamide (AC) normalised tumour vasculature and improved perfusion, but resulted in neutropenia and delayed subsequent cycles in breast cancer patients. This study combined sunitinib with docetaxel, which has an earlier neutrophil nadir than AC.

**Methods:**

Patients with advanced solid cancers were randomized 1:1 to 3-weekly docetaxel 75 mg/m^2^, with or without sunitinib 12.5 mg daily for 7 days prior to docetaxel, stratified by primary tumour site. Primary endpoints were objective-response (ORR:CR + PR) and clinical-benefit rate (CBR:CR + PR + SD); secondary endpoints were toxicity and progression-free-survival (PFS).

**Results:**

We enrolled 68 patients from 2 study sites; 33 received docetaxel-sunitinib and 35 docetaxel alone, with 33 breast, 25 lung and 10 patients with other cancers.

There was no difference in ORR (30.3% vs 28.6%, *p* = 0.432, odds-ratio [OR] 1.10, 95% CI 0.38–3.18); CBR was lower in the docetaxel-sunitinib arm (48.5% vs 71.4%, *p* = 0.027 OR 0.37, 95% CI 0.14–1.01). Median PFS was shorter in the docetaxel-sunitinib arm (2.9 vs 4.9 months, hazard-ratio [HR] 2.00, 95% CI 1.15–3.48, *p* = 0.014) overall, as well as in breast (4.2 vs 5.6 months, *p* = 0.048) and other cancers (2.0 vs 5.3 months, *p* = 0.009), but not in lung cancers (2.9 vs 4.1 months, *p* = 0.597). Median OS was similar in both arms overall (9.9 vs 10.5 months, HR 0.92, 95% CI 0.51–1.67, *p* = 0.789), and in the breast (18.9 vs 25.8 months, *p* = 0.354), lung (7.0 vs 6.7 months, *p* = 0.970) and other cancers (4.5 vs 8.8 months, *p* = 0.449) subgroups.

Grade 3/4 haematological toxicities were lower with docetaxel-sunitinib (18.2% vs 34.3%, *p* = 0.132), attributed to greater discretionary use of prophylactic G-CSF (90.9% vs 63.0%, *p* = 0.024). Grade 3/4 non-haematological toxicities were similar (12.1% vs 14.3%, *p* = 0.792).

**Conclusions:**

The addition of sunitinib to docetaxel was well-tolerated but did not improve outcomes. The possible negative impact in metastatic breast cancer patients is contrary to results of adding sunitinib to neoadjuvant AC. These negative results suggest that the intermittent administration of sunitinib in the current dose and schedule with docetaxel in advanced solid tumours, particularly breast cancers, is not beneficial.

**Trial registration:**

The study was registered (NCT01803503) prospectively on clinicaltrials.gov on 4th March 2013.

## Background

Combining the anti-angiogenic monoclonal antibody bevacizumab with chemotherapy has been shown to improve survival outcomes in various cancers [1, 2]. However, data from Phase III randomized trials evaluating the addition of small molecule anti-angiogenic tyrosine kinase inhibitors (TKIs) like sunitinib and sorafenib to chemotherapy in solid tumours have been largely negative [3–5]. One possible reason is that optimal dosing and scheduling has not yet been determined. Pre-clinical studies have suggested that anti-angiogenic agents could transiently normalize tumour vasculature, but further continuous administration at full dose of these agents results in destruction of tumour vasculature [6, 7]. This may paradoxically result in reduced delivery of chemotherapy to the tumour [8, 9]. Intermittent dosing of a small molecule TKI at a lower dose prior to chemotherapy to transiently ‘normalize’ tumour vasculature may improve drug and oxygen delivery and thus potentiate sensitivity to chemotherapy [10, 11].

We previously conducted a Phase Ib followed by randomized Phase II trial of short-course, low-dose sunitinib prior to neoadjuvant doxorubicin-cyclophosphamide (AC) for 4 cycles in breast cancer in an attempt to normalize tumour vasculature. In phase Ib, sunitinib 25 mg daily for 1 week prior to AC resulted in tumor vessel destruction on immunohistochemistry, while a lower dose of 12.5 mg daily normalized tumor vessels. Thus, 12.5 mg sunitinib for 1 week was tested in the phase II randomized trial of AC with or without pre-treatment with 12.5 mg sunitinib. Low dose sunitinib + AC resulted in immunohistochemical evidence of increased vascular normalization index, DCE-MRI evidence of improved perfusion, higher objective clinical response rates (60.9% vs 34.8%, *p* = 0.08), and tumour volume reduction measured on DCE-MRI compared to AC alone. However, the pathological complete response rate at surgery after 4 cycles of AC was not different with or without sunitinib, which we postulated may have been attributed to increased myelosuppression from sunitinib causing significantly more treatment delays in subsequent AC cycles [12].

We hypothesized that docetaxel, which has an earlier neutrophil nadir at Day 7–10 compared to AC, will result in less overlapping myelosuppression when combined with intermittent dosing sunitinib in breast cancer as well as other solid tumors, and tested this hypothesis in a randomized phase II trial.

## Methods

### Patients

We enrolled patients who were at least 18 years of age and had a histologic or cytologic diagnosis of carcinoma, with a measurable tumour (defined as a clinically palpable tumour with both diameters 2.0 cm or greater, or radiologically measurable by RECIST criteria). Patients who had advanced solid tumours that were not amenable to curative treatment and had an estimated life expectancy of at least 12 weeks were included. Other key eligibility criteria included Eastern Cooperative Oncology Group (ECOG) performance status of 0 or 1, and adequate haematologic, hepatic and renal function. Patients were excluded if they were pregnant or lactating, or had active infections, active bleeding, poorly controlled diabetes mellitus, non-healing wounds, symptomatic brain metastases, systemic connective tissue diseases or second primary malignancies. All patients gave written informed consent.

### Study design

This study was a randomized open-label Phase II trial of intermittent sunitinib in combination with docetaxel involving two study sites- the National University Cancer Institute in Singapore and the University of Malaya Medical Centre in Malaysia. Patients were randomized 1:1 to receive sunitinib and docetaxel or docetaxel alone. Randomization was stratified according to the primary tumour (breast vs lung cancer vs others) to account for the potential impact of tumor type on treatment response. Simple randomization was performed by the selection of sealed envelopes containing the designated study arm. These were kept and allocated by a research administrator who was not involved in patient enrollment or treatment.

Patients in the experimental arm received sunitinib 12.5 mg orally for 7 days prior to docetaxel. Docetaxel was given at 75 mg/m^2^, once every 3 weeks, up to a maximum of 6 cycles. In the control arm, patients received docetaxel alone. The administration of prophylactic colony stimulating factors was at the discretion of the investigators. Standard pre-medications were employed for the first cycle, with modifications in subsequent cycles permitted at investigator’s discretion. Dose modifications of docetaxel in response to toxicities were allowed. No dose modifications were allowed for sunitinib. Treatment was discontinued at the point of tumour progression, unacceptable toxicities or withdrawal of patient consent.

The study was registered (NCT01803503) and approved by the institutional review board at each participating centre and was conducted in accordance with the Declaration of Helsinki and Good Clinical Practice guidelines. The study adheres to CONSORT guidelines [13].

### Efficacy, safety

The co-primary endpoints of the study were objective response rate (defined as the proportion of patients who achieved complete or partial response) and clinical benefit rate (proportion of patients who achieved complete response, partial response or stable disease for at least 12 weeks). Secondary endpoints included progression-free survival, defined as the time from randomization to date of documented disease progression or death from any cause, and safety. If progression or death did not occur, patients were continued on follow-up and censored at the date of last contact, or the date of study closure (27 February 2020), whichever was earlier.

Tumour response was assessed according to the Response Evaluation Criteria in Solid Tumours version 1.1 (RECIST 1.1) [14]. Clinical assessments were performed at baseline (within 2 weeks prior to enrollment), and every 3 weeks, prior to the start of every treatment cycle. Radiologic assessments with CT scan of the thorax and abdomen (+/− pelvis at investigator’s discretion) were performed at baseline (within 4 weeks prior to enrollment), and prior to the start of every other treatment cycle. Response assessment was conducted by the investigator. Safety was assessed by documentation of adverse events, patient reporting, physical examination and biochemical and haematologic clinical laboratory tests. Patients were evaluated weekly for toxicity assessments during cycle 1, and on days 1 and 15 of each subsequent cycle. Adverse events were graded with the use of the Common Terminology Criteria for Adverse Events of the National Cancer Institute version 3 (NCI CTCAE v3). All patients who received at least one dose of study drug were included in the efficacy and safety analysis.

Blood samples for pharmacokinetic sampling were taken at 0 min (prior to dosing), 30 min, 60 min, 2 h, 5 h, 7 h and 24 h on Cycle 1 Day 1 for docetaxel. Docetaxel concentrations were analysed using liquid chromatography-tandem mass spectrometry [15].

### Statistical analysis

The estimated objective response rate to docetaxel in advanced solid tumours is approximately 20% [16, 17]. We hypothesized that the concomitant administration of intermittent sunitinib with standard docetaxel will increase the objective response rate by 2-fold to approximately 40% based on our previous observation that adding low-dose intermittent sunitinib to doxorubicin/cyclophosphamide chemotherapy almost doubled the objective clinical response rate after cycle 1 chemotherapy from 34.8 to 60.9% [12]. Based on a power of 80% and a one-sided test size of 15%, a minimum sample size of 33 evaluable patients per group was required.

The primary endpoints were analyzed in all patients who received the randomized treatment, in an intention to treat analysis assuming a one-sided test, whereas secondary endpoints were evaluated using a two-sided test at the 5% level of significance. Safety analyses were performed on the basis of treatment received. Response rates between treatment groups were compared using the Mantel-Haenszel method, stratified by primary tumour. Progression-free and overall survival analysis was performed using the Kaplan-Meier method, with differences in survival distributions compared using the stratified log-rank test, stratified by tumour site. A stratified Cox proportional-hazards model was used to estimate hazard ratios and 95% confidence intervals.

## Results

### Patient characteristics

**Fig. 1:**
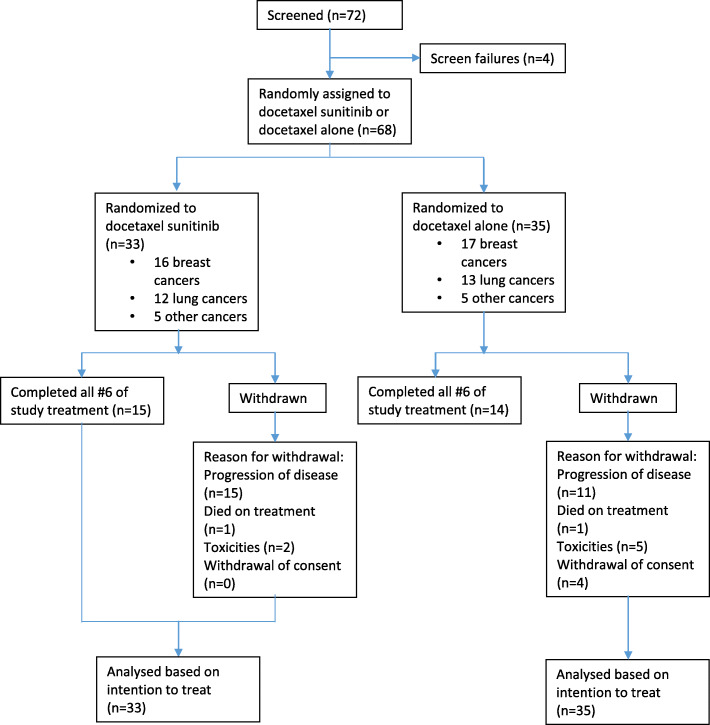
Consort diagram

Between August 2013 and September 2017, 68 patients were enrolled (49 at the National University Cancer Institute, Singapore and 19 at University of Malaya, Malaysia). Trial enrollment was closed in September 2017 as the trial had completed enrollment. Of these, 33 were randomized to the docetaxel-sunitinib arm and 35 to the docetaxel alone arm; 33 patients had breast cancer, 25 patients had lung cancer and 10 patients had other cancers (Fig. 1). The majority of patients were female (64.7%), Chinese (66.2%) and had adenocarcinoma (52.9%). The treatment groups were balanced with respect to baseline demographic and disease characteristics, except that the docetaxel-sunitinib group had a higher proportion of Chinese patients (84.8%) compared to the docetaxel alone group (48.6%) (Table 1). Of the patients in the docetaxel-sunitinib group, 33.3% had received 2 or more previous lines of therapy in the advanced setting, versus 40.0% of the patients in the docetaxel alone group, and 30.3% versus 31.4% respectively had received no previous palliative therapy.

**Table 1:** Demographic and Disease Characteristics of the Patients at Baseline

Characteristics	Docetaxel-sunitinib (*N* = 33)	Docetaxel alone (*N* = 35)	Overall (*N* = 68)
Gender			
Male	11 (33.3%)	13 (37.1%)	24 (35.3%)
Female	22 (66.7%)	22 (62.9%)	44 (64.7%)
Mean age (range), years	55.4 (34.0–76.0)	57.1 (35.7–74.5)	56.3 (34.0–76.0)
Race			
Chinese	28 (84.8%)	17 (48.6%)	44 (66.2%)
Indian	0 (0%)	3 (8.6%)	3 (4.4%)
Malay	4 (12.1%)	11 (31.4%)	15 (22.1%)
Others	1 (3.0%)	4 (11.4%)	5 (7.4%)
Site of primary tumour			
Breast	16 (48.5%)	17 (48.6%)	33 (48.5%)
Lung	12 (36.4%)	13 (37.1%)	25 (36.8%)
Others	5 (15.2%)	5 (14.3%)	10 (14.7%)
`Histology			
Adenocarcinoma	16 (48.5%)	20 (57.1%)	36 (52.9%)
Squamous carcinoma	13 (39.4%)	12 (34.3%)	25 (36.8%)
Others (including poorly differentiated, NOS, lymphoma, small cell)	4 (12.1%)	3 (8.6%)	7 (10.3%)
Lines of prior palliative therapy			
0	10 (30.3%)	11 (31.4%)	21 (30.9%)
1	12 (36.4%)	10 (28.6%)	22 (32.4%)
2	6 (18.2%)	8 (22.9%)	14 (20.6%)
3	4 (12.1%)	2 (5.7%)	6 (8.8%)
4	1 (3.0%)	2 (5.7%)	3 (4.4%)
>/=5	0 (0%)	2 (5.7%)	2 (3.0%)

### Treatment characteristics

At the time of analysis, 64 patients had progressed or died, and 48 patients had died. All 68 patients have discontinued treatment: 29 patients completed all 6 cycles of docetaxel treatment, 26 patients had treatment stopped due to progression of disease, 2 died during treatment, 7 were taken off the study due to toxicities and 4 withdrew from the study for other reasons.

The mean duration of treatment was 12.4 weeks in the docetaxel-sunitinib group and 14.1 weeks in the docetaxel alone group (*p* = 0.284). The mean dose intensity of docetaxel in the docetaxel-sunitinib arm was 23.6 mg/m^2^/week, vs 24.5 mg/m^2^/week in the docetaxel alone arm (*p* = 0.303). Patients received a median of 4 and 5 cycles of treatment in each group respectively (*p* = 0.940). Dose delays of ≥1 week were seen in 18.2 and 11.4% of patients in the docetaxel-sunitinib versus docetaxel alone groups (*p* = 0.432). Of the 49 patients in whom use of granulocyte stimulating factor (G-CSF) was recorded, 37/49 (76%) received G-CSF as prophylaxis (36 as primary prophylaxis, 1 as secondary prophylaxis). Significantly more patients in the docetaxel-sunitinib arm received G-CSF compared to those in the docetaxel alone arm (90.9% vs 63.0%, *p* = 0.024).

### Efficacy

The primary endpoint of objective response rate was similar in both arms, with an ORR of 30.3% in the docetaxel-sunitinib arm vs 28.6% in the docetaxel alone arm (*p* = 0.432, OR 1.10, 95% CI 0.38–3.18). Clinical benefit rates were lower in the docetaxel-sunitinib arm at 48.5% vs 71.4%, (*p* = 0.027, OR 0.37, 95% CI 0.14–1.01). Subgroup analysis was performed on ORR and CBR in the different tumour types. In breast cancer, the ORR was 43.8% vs 35.3% (*p* = 0.619) and the CBR 68.8% vs 76.5% (*p* = 0.619); in lung cancer, the ORR was 16.7% vs 30.8% (*p* = 0.409) and the CBR 33.3% vs 53.8% (*p* = 0.302); in other cancers, the ORR was 20% vs 0% (*p* = 0.292) and the CBR 20% vs 100% (*p* = 0.010).

**Fig. 2:**
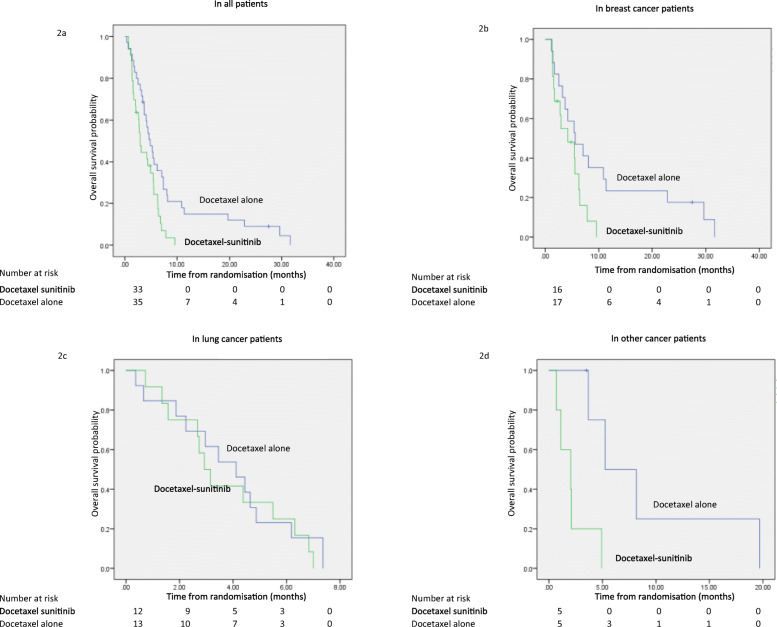
Progression-free survival 2a Progression-free survival in the overall patient population 2b Progression-free survival in the breast cancer subgroup 2c Progression-free survival in the lung cancer subgroup 2d Progression-free survival in the other cancers subgroup

**Fig. 3:**
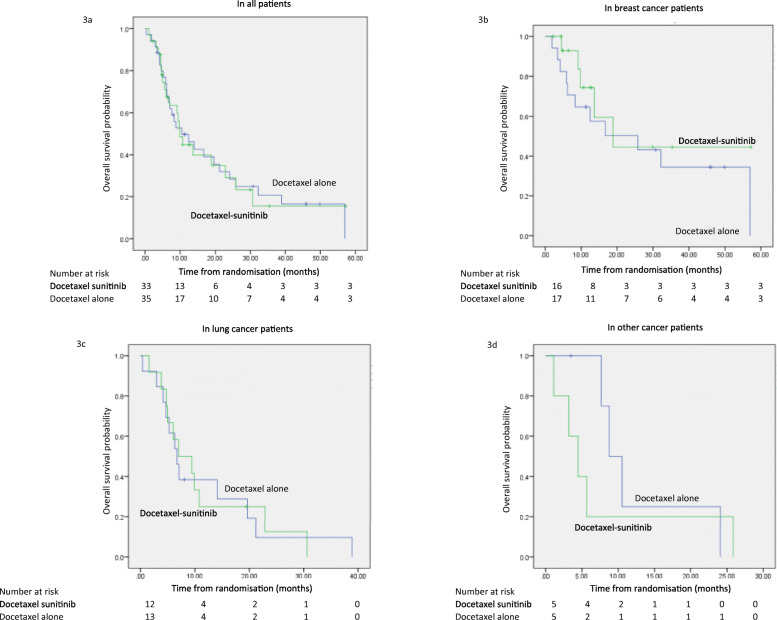
Overall Survival 3a Overall survival in the overall patient population 3b Overall survival in the breast cancer subgroup 3c Overall survival in the lung cancer subgroup 3d Overall survival in the other cancers subgroup

In the secondary endpoint of progression-free survival (PFS), the addition of sunitinib to docetaxel was associated with a shorter PFS when compared with docetaxel alone (2.9 months vs 4.9 months, HR 2.00, 95% CI 1.15–3.48, *p* = 0.014) (Fig. 2). Exploratory subgroup analysis by tumour type showed the difference in PFS remained significant in patients with breast cancer (4.2 months vs 5.6 months, *p* = 0.048) and other cancers (2.0 months vs 5.3 months, *p* = 0.009), but was not significant in the lung cancer subgroup (2.9 months vs 4.1 months, *p* = 0.597). Overall survival (OS) was similar in both groups (9.9 months vs 10.5 months, HR 0.922, 95% CI 0.51–1.67, *p* = 0.789) (Fig. 3). There was also no OS benefit from the addition of sunitinib in any of the tumour subgroups: in breast cancers, OS was 18.9 months vs 25.8 months (*p* = 0.354), in lung cancers, OS was 7.0 months vs 6.7 months (*p* = 0.970) and in other cancers OS was 4.5 months vs 8.8 months (*p* = 0.449).

### Adverse events

The addition of sunitinib to docetaxel was generally well tolerated. The rates of adverse events of all grades was similar in both arms, at 84.8% in the docetaxel-sunitinib arm versus 91.4% in the docetaxel alone arm (*p* = 0.400). Rates of Grade 3/4 non-haematological toxicities were similar in both arms, at 12.1% versus 14.3% (*p* = 0.792), but rates of Grade 3/4 haematological toxicities were lower in the docetaxel-sunitinib arm at 18.2% compared to 34.3% in the docetaxel alone arm (*p* = 0.132), likely attributed to greater use of prophylactic growth factors in the docetaxel-sunitinib arm. 0 and 5.7% of patients experienced febrile neutropenia respectively (*p* = 0.163). None of the dose delays were due to neutropenia; reasons for delay included exacerbation of chronic obstructive pulmonary disease, drainage of pleural effusions and herpes zoster. Table 2 shows the rates of serious toxicities and all grade toxicities that occurred in ≥5% patients. As expected, low dose, short course sunitinib was not associated with VEGFR tyrosine kinase inhibitor related adverse events.

**Table 2:** Adverse Events

Toxicity	All grades			Grade 3 to 5		
	Docetaxel-sunitinib (*N* = 33)	Docetaxel alone (*N* = 35)	*P* value	Docetaxel-sunitinib (*N* = 33)	Docetaxel alone (*N* = 35)	*P* value
All toxicities	84.8%	91.4%	0.400	30.3%	42.9%	0.285
Fatigue	30.3%	45.7%	0.145	6.1%	0%	0.232
Peripheral neuropathy	27.3%	31.4%	0.457	0%	2.9%	0.515
Diarrhoea	27.3%	25.7%	0.551	0%	0%	NA
Neutropenia	24.2%	31.4%	0.509	18.2%	31.4%	0.207
Nausea	24.2%	22.9%	0.559	0%	0%	NA
Anorexia	18.2%	20.0%	0.547	0%	0%	NA
Vomiting	12.1%	14.3%	0.539	0%	0%	NA
Fever	12.1%	11.4%	0.611	6.1%	11.4%	0.435
Cough	12.1%	8.6%	0.466	0%	0%	NA
Xerostomia	12.1%	2.9%	0.160	0%	0%	NA
Dizziness	12.1%	0%	0.050	0%	0%	NA
Myalgia	9.1%	22.9%	0.112	0%	0%	NA
Dyspnoea	6.1%	17.1%	0.149	0%	2.9%	0.515
Dyspepsia	6.1%	14.3%	0.239	0%	0%	NA
Mouth ulcers	6.1%	11.4%	0.365	0%	0%	NA
Insomnia	6.1%	8.6%	0.528	0%	0%	NA
Hypersensitivity	6.1%	5.7%	0.670	0%	0%	NA
Herpes Zoster	6.1%	2.9%	0.478	3.0%	0%	0.485
Sore throat	6.1%	2.9%	0.478	0%	0%	NA
Dysgeusia	3.0%	5.7%	0.522	0%	0%	NA
Lower limb oedema	3.0%	5.7%	0.522	0%	2.9%	0.515

### Pharmacokinetic analysis

Of the 68 patients enrolled in the study, 63 had blood samples available for pharmacokinetic analysis (Table 3). Docetaxel area under the curve (AUC_0-∞_) was numerically higher for patients who received docetaxel-sunitinib vs those with docetaxel alone (6.48 ± 4.43 h*μg/ml vs 4.85 ± 1.76 h*μg/ml, *p* = 0.053), although the difference did not reach statistical significance. Docetaxel clearance was not significantly different in the two groups (24.4 ± 13.2 L/h vs 27.4 ± 8.7 L/h for docetaxel-sunitinib vs docetaxel alone, *p* = 0.290). The predicted volume of distribution of docetaxel (Vss_pred) was similar in both arms at 193.5 ± 248.2 L vs 198.5 ± 148.6 L (*p* = 0.922).

**Table 3:** Docetaxel Pharmacokinetics (*n* = 63)

	Docetaxel-sunitinib	Docetaxel alone	*P* value
Tmax (h)	0.95 ± 0.43	0.76 ± 0.33	0.060
Cmax (μg/ml)	4.28 ± 2.07	3.40 ± 1.26	0.044
AUC_0-∞_ (hours*μg/ml)	6.48 ± 4.43	4.85 ± 1.76	0.053
Cl_pred (L/h)	24.4 ± 13.2	27.4 ± 8.7	0.290
Vss_pred (L)	193.5 ± 248.2	198.5 ± 148.6	0.922

Tmax time to peak serum concentration; Cmax maximum serum concentration; *AUC0-*∞ area under concentration-time curve; Cl_pred predicted clearance; Vss_pred predicted volume of distribution at steady state

We examined whether docetaxel AUC_0-∞_ correlated with efficacy outcomes. Using mean AUC_0-∞_ as the cutoff to divide the cohort into those with high versus low docetaxel AUC_0-∞,_ no significant difference in PFS, objective response rate, or clinical benefit rate, was observed between patients with high versus low docetaxel AUC_0-∞_ (PFS 4.1 months vs 4.6 months, *p* = 0.141; ORR 30.4% vs 25.0%, *p* = 0.640; CBR 56.5% vs 62.5%, *p* = 0.641).

## Discussion

We have previously shown that administering low-dose, short-course sunitinib at 12.5 mg for 1 week before neoadjuvant doxorubicin-cyclophosphamide (AC) chemotherapy in breast cancer resulted in histological evidence of vascular normalization and DCE-MRI evidence of improved tumour perfusion and greater reduction in tumour volume, possibly due to improved chemotherapy delivery into the cancer. However, this did not translate to improved rates of pathologic complete response after repeated cycles of chemotherapy. We postulated that this could be due to more prolonged neutropenia from AC chemotherapy with addition of sunitinib that led to overall reduced chemotherapy dose intensity [12].

We therefore set out to evaluate the same strategy of low-dose, short-course sunitinib preceding chemotherapy but with docetaxel, which has an earlier neutrophil nadir than AC, with the hypothesis that this combination will be less myelosuppressive. Disappointingly, we observed no improvement in objective response rate, clinical benefit rate, or progression-free survival with the addition of sunitinib. One possible reason is that the normalization of tumour vasculature achieved with sunitinib may be insufficient to translate to a clinically significant difference. Another possible reason is that our current dose and schedule of sunitinib is not the clinically optimal regimen to normalize tumour vasculature in combination with docetaxel. In our previous neoadjuvant AC-sunitinib trial, we observed normalization of tumour vasculature to occur as early as 24 h after treatment, with the most significant increase in vascular normalization index occurring after the first cycle of chemotherapy, but only modest further increase with subsequent treatment cycles. This study and others underscore the sensitivity of tumour vasculature to dosing and scheduling of anti-angiogenic agents. In this current trial, we administered intermittent dosing sunitinib with up to 6 cycles of docetaxel, but did not observe any improvements in response rates compared to docetaxel alone. We hypothesize that while initial treatment with sunitinib does normalize tumour vasculature, repeated administration may conversely compromise normal tumour vasculature and eventually impair chemotherapy delivery. In fact, it may be possible that just a single cycle or two of sunitinib prior to starting chemotherapy may be sufficient to normalise tumour vasculature, but this is a question that will have to be addressed by a differently designed clinical trial. It is also uncertain as to whether the different mechanisms of action of AC (combining an intercalating and an alkylating agent) and docetaxel (which inhibits microtubule disassembly) act synergistically with sunitinib to varying extents, however, we do not expect this factor to have an impact on the results of our study.

Surprisingly, progression-free survival was actually significantly lower in patients who received sunitinib for the entire cohort. While this was a secondary endpoint of the trial and clear conclusions on survival cannot really be drawn, this result was nonetheless unexpected. The observed difference in PFS is unlikely to be due to differences in patient characteristics, which were well matched down to the number of previous lines of therapy, and primary tumour type. It is also not likely to be attributable to differences in toxicities, as toxicity rates were similar in both arms. There were more dose delays in patients on the docetaxel-sunitinib arm, despite the fact that there was a higher rate of growth factors administered in this arm; however, dose intensity of docetaxel administration was similar in both arms and again unlikely to account for the inferior PFS seen in those patients.

Interestingly, a Phase II trial that studied the addition of sunitinib 37.5 mg daily to pemetrexed versus pemetrexed alone in the second line treatment of NSCLC has similarly shown detrimental effects on overall survival, although progression-free survival was not significantly different [18]. Multiple Phase III trials have added sunitinib or sorafenib to first-line chemotherapy in breast, lung and colorectal cancers, all of which did not result in poorer PFS [3–5]. One potential reason is that these trials, as well as our prior neoadjuvant AC-sunitinib trial, evaluated relatively treatment-naïve patients receiving first line systemic therapy, whereas patients in our current trial and the negative Phase II lung cancer trial were more heavily pre-treated, suggesting that tumours that have had prior exposure to systemic therapies may be more resistant to the vasculature normalization effect of low dose sunitinib.

We noted on subgroup analysis that while PFS was significantly worse with the addition of sunitinib in breast and other cancers, this was not the case with the lung cancer subgroup. This observation is concordant with reported Phase III trials in non-squamous non-small cell lung cancers, where the addition of bevacizumab to carboplatin-paclitaxel, or the addition of ramucirumab or nintedanib to docetaxel, all showed survival benefits, in contrast to breast cancer studies on addition of bevacizumab to chemotherapy [2, 19–22]. This could potentially be due to differences in tumour vasculature and expression or influence of angiogenic growth factors in the different tumour types, in turn affecting response to anti-VEGF agents. In future studies, collection of tumour tissue for analysis of angiogenic growth factors such as VEGF receptors or Hypoxia Inducible Factor 1 Alpha (HIF1 Alpha) could help to shed light on this issue.

We observed that docetaxel AUC is higher in patients who received docetaxel with sunitinib than those who received docetaxel alone. Both docetaxel and sunitinib undergo hepatic metabolism by the cytochrome P450 CYP3A4 system, and we postulate that the concomitant administration of both drugs may slow docetaxel metabolism and excretion, leading to a higher docetaxel AUC. Yet despite this higher docetaxel AUC, we do not see corresponding improvements in response rate or increases in toxicity. PFS and response rates also did not correlate with docetaxel AUC.

Our study has several limitations. The sample size was small, patients were recruited from only two institutions, and treatment was not blinded. In addition, the study recruited patients with a variety of solid tumors rather than focused on a single tumor type. Although we had initially hypothesized that different solid tumors will have broadly similar response to the therapeutic strategy of low-dose, short-course sunitinib to improve chemotherapy delivery and efficacy, we did stratify patients by tumor type before randomization and found preliminary evidence from this study that there is indeed heterogeneity in responses between different tumor types. Finally, overall survival follow-up is immature for breast cancer patients in our trial, with 6 of 33 patients still alive at data-cutoff of 5 years.

## Conclusions

In conclusion, the addition of low-dose, short-course sunitinib to docetaxel chemotherapy is well tolerated, but did not improve objective response or clinical benefit rates in advanced solid tumours. These negative results suggest that the intermittent administration of sunitinib in the current dose and schedule with docetaxel in advanced solid tumours, particularly breast cancers, is not beneficial. If this strategy were to be explored further, then additional studies will be warranted to determine the optimal regimen of anti-angiogenic agents in combination with chemotherapy to achieve improved drug delivery, to ascertain if pathologic tumour vasculature changes can translate into meaningful clinical benefit, and to identify groups of patients most likely to benefit from this strategy.

## Data Availability

The datasets generated during and/or analysed during the current study are available from the corresponding author on reasonable request.
